# Environmental and social-motivational contextual factors related to youth physical activity: systematic observations of summer day camps

**DOI:** 10.1186/1479-5868-10-63

**Published:** 2013-05-20

**Authors:** Nicole Zarrett, Carl Sorensen, Brittany Skiles

**Affiliations:** 1 Department of Psychology, University of South Carolina, 1512 Pendleton Street, Columbia, SC, 29208, USA

**Keywords:** Physical activity, Summer camp, Children and adolescents, Obesity, Assessment tools, Self-determination theory, Systematic observation

## Abstract

**Background:**

Youth risk of obesity is high during the summer months. Summer day camps can be ideal settings for preventing obesity through reducing youth summer sedentary behaviors. However, with limited research on camp settings, the mechanisms by which these programs promote children’s physical activity (PA) remains largely unknown. The current study was designed to take a first step in addressing this gap in research through systematic observations of 4 summer day camps.

**Methods:**

Systematic observations of 4 summer day camps was conducted using the System for Observing Play and Leisure Activity in Youth (SOPLAY) and a social-motivational climate supplemental observation tool founded on Self-Determination Theory and previous research developed by the authors. Teams of two coders observed daily activities for four days across two-week periods at each camp. On 15 minute intervals throughout each day, camps were assessed on level of youth PA (e.g., sedentary, moderate, vigorous), five physical features (e.g., equipment), eight staff interactions (e.g., encourage PA), and six social climate components (e.g., inclusive game).

**Results:**

Across the sample, highly engaging games [F(1,329) = 17.68, p < .001], positive peer interactions [F(1,329) = 8.43, p < .01], and bullying [F(1,329) = 9.39, p < .01] were significantly related to higher PA participation rates, and clarity of rules [F(1,329) = 11.12, p < .001] was related to fewer youth participating in PA. Separate analyses for males and females indicated some sex differences with highly engaging games [F(1,329) = 23.10, p < .001] and bullying [F(1,329) = 10.00, p < .01] related to males’ but not females’ PA, and positive peer interactions related to only females’ PA [F(1,329) = 9.58, p < .01]. Small, yet significant physical-environmental effects of temperature [F(1,328) = 1.54, p < .05] and equipment [F(1,328) = 4.34, p = .05] for girls also suggests that activities offered indoors (which was most common during high temperatures), and provision of equipment may also be important considerations for promoting girls’ PA. Staff behaviors were minimally predictive of youth PA.

**Conclusions:**

This is the first study to conduct systematic observations of the physical and social resources of summer day camps and contributes to our understanding of the strengths and needs of camps to effectively promote PA in both boys and girls during the summer months when risks for obesity are high.

## Background

Lack of physical activity (PA) has been identified as a primary cause for the pervasiveness of childhood obesity worldwide [1-5]. Although there has been considerable focus on interventions to increase youth PA in afterschool programming throughout the school year [6,7], there has been much less research on ensuring that youth participate in adequate amounts of PA during the summer months when the majority of schools within the U.S., Europe, and Canada, have an 8-to-14 week summer break from school [8]. Despite popular notions of summer as a highly active time for youth, some research has indicated the rate of children’s BMI increase during summer is more than double the rate during the school year [9].

Although research is still needed to fully understand summer weight gain and its contributors across a diversity of youth [10], some researchers have speculated that the summer break from school may result in less structured days for youth leading to months of less physical activity and a less healthy diet [9,11-13]. In turn, summer day camps, which typically offer a variety of structured and guided PA opportunities for extended periods of time (e.g., 6–8 hrs/day for 8 weeks), make it a promising setting for establishing and reinforcing youth healthy behaviors and may be a critical resource for prevention of obesity and related disease [14].

Within the U.S., camp programs serve an estimated 12 million campers each year [15]. Similar to the community-based youth development programs offered afterschool (e.g., Boys & Girls Club, 4-H), the mission/curriculum of most summer day camp programs are founded on the theoretical foundations of Positive Youth Development (PYD) [16] which, among its goals, includes a focus on providing supports and opportunities necessary to promote the healthy physical development of participating youth [17]. However, with limited research on summer day camp settings [18,19], the mechanisms by which these programs promote youth healthy behaviors (e.g., youth PA) remains largely unknown. Specifically, no studies to-date have measured what physical (e.g., access to a playground, equipment) and social-motivational (e.g., challenging and inclusive games, staff encouragement and participation) features of the camp setting are most effective for promoting youth PA. The current study was designed to take a first step towards addressing this gap in research through systematic observations of 4 summer day camps (2 highly- resourced and 2 low-resourced camps). Given previous research findings on sex differences in what motivates youth to participate in PA [20-25], similarities and differences in how context features were related to boys’ and girls’ PA was also considered in the present study.

### Mechanisms for promoting youth PA in camp settings

Along with the built environment [26-28], evidence suggests that social environments can be manipulated to increase and maintain youth PA [29-31]. According to Self Determination Theory (SDT) [32], effective programs for fostering youth intrinsic engagement in PA are social-motivational contexts: 1) that provide challenging experiences that interest youth and where youth can demonstrate their abilities (competence); 2) where youth feel accepted and a sense of belonging (relatedness), and; 3) where it is safe to share their ideas, explore their identities and interests, and where behavior is self-determined and not guided by external incentives (i.e., autonomy). Previous PA-based interventions indicate the integration of perceived choice, self-initiated behaviors, and sense of belonging, are instrumental in increasing youth intrinsic motivation, effort, and persistence for engaging in PA [33-36]. Perceived competence, autonomy, enjoyment, social connectedness, and degree of outside pressure have also been identified as primary motives for engaging in physical education (PE) [30,37,38], and for pursuing or dropping out of sports [39-43].

Lastly, research has demonstrated that staff behaviors and positive staff-child interactions may be essential for meeting youth motivational needs and, in turn, promoting youth intrinsic engagement. In particular, numerous studies have shown that an autonomy supportive, well-structured, and interpersonally-involving interaction style among staff/teachers was predictive of youth greater motivation and participation in PA [44-49].

Although decades of education research has used well-established observational assessment tools to highlight the importance of the social-motivational climate of school classrooms for promoting youth engagement [50-55], little attention has been focused on systematic observational assessment of the physical and social climate of out-of-school youth institutions such as summer camps. A study by Coleman and colleagues (2008) is the only research to date that assessed the quality of after-school programs for promoting PA (without intervention) using momentary sampling behavioral observation methods [56]. Contrary to previous school-based studies which suggested structured activities were key for promoting youth PA [57,58], findings from the afterschool study indicated youth were more active in free play than during structured activities. Consequently, Coleman et al. provided recommendations for afterschool programs to increase free play opportunities, provide more staff training in simple, structured games (to minimize management time), and improve staff use of promotion and modeling strategies for promoting youth PA.

However, despite the commonality of camps and afterschool programs as recreationally-based opportunities that (should) meet the psychosocial and developmental needs of youth, their structure and focus are considerably different. Community-based afterschool programming (PYD programming) is typically focused on promoting academic success with much less time devoted to PA opportunities. Moreover, much of the time allotted to PA consists of unstructured ‘free play’ where youth can chose among a variety of active (e.g., basketball) and non-active (sit and socialize, play video games) activities in which to participate [59]. In contrast, camps are primarily focused on active recreational activities which are typically organized and varied. Summer day camps spend minimal time on academic activities (unless academically–based like “Summer Learning Programs”), are typically more staffed than afterschool programs, and youth spend an extensive amount of daily time at the program (approx 6–8 hours) which is believed to increase impact of participation in the program [15,60]. Given these significant differences in mission, structure, curriculum, and dose, we are unclear of whether the same recommendations for afterschool programs would apply to summer camps.

Moreover, no observational study to date has considered SDT-based motivational climate components in the assessment of afterschool or summer camp settings for promoting PA [60]. Similar to youth engagement in school classrooms and aligned with SDT, youth engagement in camp activities is likely to be highly dependent on the physical environment (e.g., access to equipment), the subject matter and how it is delivered (e.g., inclusive, challenging, involves youth autonomy), the behaviors and attitudes of the counselors delivering it (e.g., responsive, supportive, focused on mastery), and characteristics of program youth [36,62-65].

In the present study, an observation tool designed by the authors and founded on the theoretical foundations of SDT was used to assess the relation between physical and social-motivational characteristics of camp settings and youth engagement in PA (see Table 1 for a detailed description of program features assessed). Educators and applied behavioral analysts refer to this approach as ecobehavioral assessment, and in education research it has been shown to provide an important heuristic for methodologically identifying situational factors that either promote or impede the occurrence of specified student behaviors during subsequent school years and grade levels [52].

**Table 1:** Constructs of the SOPLAY and MCOT-PA systematic observational assessment

**Camp context constructs**	**Description**
**Conditions**	**Physical conditions of the facility for PA**
Accessible	Youth are able and allowed in the space (e.g., door unlocked)
Usable	Area is usable for PA (sufficient space, not too wet or windy)
Supervised	Program staff are present
Organized	Organized PA is being held in the space
Equipment	Removable PA equipment is available (e.g., balls, jump ropes)
**Activity**	**Levels of youth PA**
Sedentary	(e.g., lying, sitting, standing still)
Walking	(e.g., walking, shifting weight from foot to foot)
Vigorous	(e.g., running, sit ups, climbing, etc.)
**Climate**	**Youth and Activity components**
Clarity of Rules	Youth understand activity rules and are able to follow them
Autonomy/Choice	Youth have opportunities to make choices and voice opinions (e.g., activity options are available, participation is not mandated)
High Engagement	Activity is optimally challenging and fun (e.g., skill level appropriate; youth are smiling, squealing, laughing or “in the zone”)
Inclusion	Most youth are allowed, able, and willing to participate in the activity (e.g., no youth are discouraged from participating, the majority of youth are interested and participate)
Positive Interactions	Youth demonstrate enjoyment interacting with peers (e.g., helping each other, working together as a team, encouraging one another)
Bullying	Youth demonstrate negative verbal and/or non-verbal interactions with peers (e.g., pushing, yelling, teasing)
**Interaction**	**Staff components**
Promotes PA during program	Staff prompts or directs PA (e.g., “roll the ball, don’t bounce it”, “go ahead”)
Increases activity engagement	Staff encourages increased intensity of PA (e.g., “go, go”, “hustle”)
Praises or reinforces PA	Staff uses verbal or physical praise to encourage PA (e.g., “nicely done on that move”, gives a high five)
Promotes *out-of-program* physical activity, fitness, or motor skills	Staff reminds or encourages PA outside of the program (e.g., practice that skill at home, you can play this game with your neighbors)
Other-task (disengaged)	Staff is disengaged (e.g., on their phone, back turned to youth while talking to someone else)
Demonstrates/Participates in fitness	Staff models PA behavior (e.g., shows a new skill, plays game with youth)
Observes	Staff watches youth activity
General Interaction	There IS staff engagement, but it is not related to PA (e.g., management)

## Methods

### Setting/participants

Four youth recreational summer day camps located within the greater Columbia area of South Carolina were targeted to participate in the project. All camps were located within a 10-mile radius of an urban center and each camp self-identified as an urban or suburban site. Although one camp was located in a region of the state that is classified as rural by typical population-based classification schemes, this camp was located within an urban, downtown center of an otherwise rural county and self-identified as “urban” with easy access to health care (major hospital less than 1mile away), restaurants (including fast food), and shopping centers. Camps were recruited from 5 widely accessible national youth programming organizations within the U.S. (Boys and Girls Club, YMCA, JCC, 4-H, and City-Sponsored Parks and Recreation Services). Recruited programs varied in resources and funding (e.g., highly resourced vs. low resourced facilities, expensive vs. inexpensive enrollment fees, variations in the diversity of youth each program serves) to further increase the ability to specify the degree to which findings could be generalized across PYD-based camp settings. Additional inclusion criteria for the camps were as follows: 1) full day camps (8-4 pm); 2) offered all summer; 3) included a physical activity component as part of the curriculum (e.g., indoor and outdoor recreation), and; 4) was founded on a PYD framework (e.g., included a program mission and daily curriculum centered on fostering physical, psychological, and achievement-related well-being rather than a specified set of skills such as “improving basketball skills”). Assessments of whether camps met the inclusion criteria were initially conducted through visiting the program websites. Conversations with program directors further confirmed whether the program met the criteria. One camp organization did not meet the inclusion criteria (programs within the greater Columbia area consisted of one week camp programs with specified themes, and some overnight camp options) and was not pursued for data collection.

Program Directors from each of the 4 organizations were approached about participation in the study and either chose a particular camp site (2 camps) or allowed us to choose one of their sites (2 camps) to observe. All recruited programs agreed to participate in the study and received a $200 honorarium for their participation. Two of the day camps were considered low-resourced camps, having less resourced facilities and equipment, minimum enrollment fees ($50-$60 per week) and serving a primarily underserved youth population defined by both minority status (91%) and low socioeconomic status (SES; 90% on free or reduced lunch). Both camps were at their maximum enrollment of 50 youth and all participating youth were observed as part of the current study (maximum observed N at Camp 1 = 54; Camp 2 = 47). Although both camps provided resources for children and adolescence from K through 12th grade (Age ranges: Camp 1 = 5 to 17 years old; Camp 2 = 6 to 12 years old) the majority of youth (Camp 1 = 77%, N = Camp 2 = 98%) were between 7 and 12 years old (*M* = 10 years old; 84% African American, 9% European American; 7% Hispanic or other). Females were slightly underrepresented in both Camp 1 (44%; N = 24) and Camp 2 (43%; N = 20).

The other two day camps were considered high resourced camps with state-of-the-art equipment, indoor and outdoor playgrounds, large outdoor areas, and considerably more expensive enrollment fees ($175-$225 per week). These camps served a primarily middle-class sample of youth from ages 6 through 12 years old. Maximum enrollment was larger for the high-resourced camps than the low-resourced camps (Camp 3: N = 120; Camp 4: N = 263), and thus, youth were separated into groups by age, with their own counselors and schedule of activities. For Camp 3, observations were conducted with all age groups except K-1 (Grades 2–6: maximum N observed = 56; *M* = 8.5 years old; 40% minority status). For Camp 4, the camp director assigned us a specified group of youth in Grades 3 and 4 to observe throughout the daily activities (maximum N observed = 38; *M* = 8 years old; 45% minority status of observed youth). Females were underrepresented in the samples observed in both Camp 3 (N = 20; 36%) and Camp 4 (maximum female N = 15; 39%). Although the number of youth at each of the four camps would vary from day-to-day, all camps required youth to enroll in the program in late spring and the majority of campers attended the program daily. Thus, the same group of youth were observed across the observation period.

Staff of all camps were hired employees that were required to attend some form of organized training prior to the start of the summer program. However, training commitments varied by camp with Camp 3 requiring a six hour training over two days, Camp 4 requiring a 40-hour training over a 3-day weekend, and both Camp 1 and Camp 2 requiring a week-long training which included 3 days of “shadowing” a senior counselor. The majority of staff at Camp 1 and 2 were employed as staff of both the afterschool and summercamp programs and had been working for the organization for an extensive period of time (ranging from 1 year to 17 years). The majority of staff at the high-resourced camps were hired seasonally with a few returning from the previous summer. Demographic data (e.g., age, race, education) were not collected on staff for the present study. Ethical approval of the study protocol was granted by the University of South Carolina's Office of Research Compliance Institutional Review Board (Pro00010833).

The present study was specifically interested in examining community-based PYD Programs (commonly referred to as “traditional” camp programs), which share fundamental commonalities in program missions and curriculum. The curriculum of all camps included active recreational experiences, time allotted for lunch and snacks, an optional arts and crafts component, and field trips. Moreover, all camps had periods within the daily curriculum where youth could choose from multiple activity options offered, and in some camps, could rotate between activities based on their interests. However there were also activities in which all youth were expected to participate (or, if uninterested, could sit out and watch). Despite these similarities, programs still varied widely on a number of dimensions, including the physical setting, targeted youth population, and the types and frequency of activities. For instance, all camps included opportunities to swim, but the two high-resourced camps had a pool on the premises and children had the opportunity to swim each day. The low-resourced camps did not have a pool on the premise and instead took weekly field trips to an outdoor community pool. Thus, youth of low-resourced camps swam less frequently. All camps allotted a large portion of their day to playing field (or gym) games, however, camps varied in the number (i.e., variety) of games offered, and the degree to which these games were organized and guided by staff. In the present study, momentary sampling observation methods was used to examine the similarities and variations in key camp characteristics in relation to youth PA.

### Procedure

Using the System for Observing Play and Leisure Activity in Youth (SOPLAY) tool and protocol [66,67], and the motivational climate supplemental observation tool designed by the authors, teams of two coders observed daily activities (e.g., sports, lunch, field trips, crafts, free play) at each camp for 4 full days across a two week period (average number of program hours observed = 17 hours, range = 16 to 18 hours; average # of observations each day =19). Aligned with previous research [56] and the SOPLAY protocol [66,67], each observation was on a different day of the week (for all camps Friday was the least attended camp day and therefore was always designated as either a practice day or excluded) and spanned a two week period to improve the likelihood of acquiring a more comprehensive account of the varied activities that take place in the camp setting. Observation scans were conducted at 15 minute intervals throughout each day of observation. Each scan assessed level of youth PA (e.g., sedentary, walking, vigorous), type of activity offered (e.g., roller skating, basketball), physical features of the setting (e.g., equipment availability), eight staff interaction components (e.g., encourages child), and six social climate components (e.g., inclusion, clarity of rules) (see Table 1). By assessing all PA and context characteristics as part of one scan we were able to capture the activity of youth and the climate as they co-occur.

Observer training and the establishment of inter-observer reliability consisted of 4 lab sessions, 2 field trials, and 1 practice day at each camp. The lab session used videotaped situations for practice, discussion, and memorization of data collection procedures and protocols. Once the team established comfort with the observational tool and reached adequate reliability with the videotaped scenarios, a two-week field trial was conducted in an afterschool program site to practice and test the feasibility of assessing the numerous program features addressed by the observation tool. After this trial run, minor modifications were made to the observation tool (e.g., changing format of observation form, collapsing codes) to improve the utility of the tool and a second 7-day field trial was conducted within a summer camp setting. During this trial run, the first author and observers discussed any discrepancies in coding. Lastly, to ensure continued reliability throughout the study, one day at each camp was dedicated to practice observations where the observers would map out the physical layout of the site, get familiar with the program ‘curriculum’, and practice observations in the new setting. Teams of two coders were used to collect all observation data. Reliability was determined by calculating inter-observer percent agreement (IOA) across all active data collection days using the formula: [(total no. agreed/total no. observed) × 100]. Interrater reliability (*r*) of .97 across all coding pairs indicated high levels of agreement. For the present study, we report findings from data collected from these systematic observations.

### Study measures

#### PA observations

Each program session was evaluated for the potential for PA using the System for Observing Play and Leisure Activity in Youth (SOPLAY) [66,67]. SOPLAY was designed to obtain observational data on the number of students and their physical activity levels during play and leisure opportunities in a specified activity area. For the present study, area scans/observations were recorded for youth PA (e.g., sedentary, walking/moderate, vigorous), area accessibility, area usability (e.g., not excessively wet), presence of supervision (e.g., camp counselor), presence of organized activity, and equipment availability (e.g., balls, jump ropes). Temperature (highest recorded for the day) was also recorded for each observation day. Although no field-based validity study of the SOPLAY measure has been conducted, validity of the activity codes used by SOPLAY have been established through heart rate monitoring [68,69].

#### Observation of the camp motivational context

The social-motivational climate of summer camps was assessed using the Motivational Climate Observation Tool for Physical Activity (MCOT-PA), an extension of the SOPLAY protocol that was developed by the authors. The MCOT-PA includes 8 staff interaction components, and six climate components to assess key social contextual features of youth settings derived from previous research and the theoretical foundations of PYD and SDT. For example, the climate components assess the degree to which the setting: 1) involves activity choices which emphasize cooperative team-based goals (inclusion) and/or engage all youth; 2) provides challenging, mastery-focused activity opportunities (e.g., high engagement), and; 3) incorporates students’ choice and input (e.g., autonomy). The staff interaction component assesses staff behaviors that foster youth engagement and cooperative play, encourage and assist youth to feel competent in the activity, and allow all youth to have input and feel respected and valued in the process. See Table 1 for a description of all SOPLAY and MCOT-PA constructs.

### Statistical analyses

Descriptive statistics were used to summarize proportion of observations in which youth were participating in sedentary, moderate, and vigorous PA, and the percentage of instances in which PA-based climate features (physical, social-motivational, and staff behavioral) were observed. Chi Square analyses and adjusted standardized residuals were used to assess the degree to which each camp setting had a significant overrepresentation or underrepresentation of instances in which PA climate features were observed. A series of Univariate Analysis of Variance models (ANOVA) were conducted to examine the relation of the physical environment, social-motivational climate features, and staff behaviors, to observed levels of youth PA. Given observations were nested within 4 camp settings, differences in variability between camps was controlled for in all analyses.

## Results

### Physical activity

Across camps, youth were sedentary for 72% of the observations, with the remaining 28% of instances involved in moderate-to-vigorous (MV)PA (moderate = 13.8%; vigorous 13.8%). Males were somewhat more active than females (Male MVPA = 30%; Female MVPA = 23%). Analysis of variance indicated differences in proportion of MVPA by camp where high resourced camps had significantly higher levels of MVPA than low resourced camps [F(1, 328) = 9.01, *p* < .01]. Differences were largely due to variations in males MVPA across camps [F(1, 328) = 12.02, *p* = .001], where the proportion of males engaged in MVPA within low resourced camps was half that of males in high resourced camps. Females MVPA did not significantly differ by camp resources.

### Mechanisms for promoting youth PA

#### Physical environment

Assessment of the physical features of the camp setting indicated that areas dedicated to supporting PA were highly accessible (100%) and usable (100%) across camps. The majority of these areas were also supervised by program staff (95%) when youth were present and provided youth PA equipment (61%; e.g., jump ropes, balls) in addition to the “built” structures of the camp site (e.g., pool, basketball hoops). Average outdoor temperatures during the time that each camp was assessed were similar and ranged from 78–96 degrees Fahrenheit (*M* = 88.5 degrees Fahrenheit; see Table 2). Chi Square analyses indicated minimal difference in the proportion of observed instances of physical resources across camps, with only significant variations found between the two low-resourced camps in the likelihood of observing the use of PA equipment [*χ*^2^ (3,328) =13.29, *p* = .004].

**Table 2:** Percentage of observed instances of physical, social climate, and staff interaction components by Camp

	**Percentage (%) of total observations**				
	**Under-resourced**		**Resourced**		
**Constructs**	**CAMP1**	**CAMP2**	**CAMP3**	**CAMP4**	**Across camps**
Physical Features					
Accessible	99.2	100	100	100	99.7
Useable	99.2	100	100	100	99.7
Supervised	95.0	91.1	100	95.2	95.1
Equipment^a^	52.1*	77.2*	62.3	56.5	61.1
Temperature^+^	89.2	86.6	87.1	91.3	88.5
Social Climate					
Clarity of Rules^b^	13.4*	46.8*	23.2	16.1	24.0
Autonomy^c^	66.4	45.6*	72.5	82.3*	65.7
Organized PA^d^	27.7	46.8*	17.4*	11.3*	27.1
High Engagement	24.4	19.0	36.2	21.0	24.9
Inclusion	20.2	13.9	29.0	16.1	19.8
Positive Youth Interactions^e^	6.7*	15.2	27.5*	12.9	14.3
Bullying	1.0	0	0	0	0.3
Staff Behaviors					
Promotes PA (Initiates)	0	2.5	4.3	0	1.6
Promotes Increases in PA	0	0	1.4	0	0.3
Praises PA	0	0	0	0	0
Promotes Outside PA	0	0	0	0	0
Other-Task (disengaged)	3.0	2.5	1.4	1.6	2.3
Demonstrates Fitness	9.0	15.2	5.8	11.3	10.3
Observes^f^	43.0*	58.2	52.2	77.4*	55.8
General interaction	45.0	21.6	34.9	9.7	31.3

^+^Temperature was reported as the mean across the 4 days of observations for each camp and the mean temperature across all camps.

a. *χ*^2^ (3,328) = 13.29, *p* = .004. Underrepresented in Camp 1 [Adjusted standardized residual (ASR) = −2.5]; Overrepresented in Camp 2 (ASR = 3.4).

b. *χ*^2^ (3,329) = 31.97, *p* = .000. Underrepresented in Camp 1 (ASR = −3.4); Overrepresented in Camp 2 (ASR = 5.4).

c. *χ*^2^ (3,329) =23.16, *p* = .000. Underrepresented in Camp 2 (ASR = −4.3); Overrepresented in Camp 4 (ASR = 3.1).

d. *χ*^2^ (3,329) =26.77, *p* = .000. Underrepresented in Camp 3 (ASR = −2.0) and Camp 4 (ASR = −3.1); Overrepresented in Camp 2 (ASR = 4.5).

e. *χ*^2^ (3,329) =15.60, *p* = .001. Underrepresented in Camp 1 (ASR = −3.0); Overrepresented in Camp3 (ASR =3.5).

f. *χ*^2^ (3,310) = 18.95, *p* = .000. Underrepresented in Camp 1 (ASR = −3.1); Overrepresented in Camp 4 (ASR = 3.8).

Although there was minimal variance in many of the physical features observed, analyses controlling for variations by camp indicated the presence of equipment (e.g., balls, jump ropes) [F(1, 328) = 4.53, *p* < .05], and higher temperatures [F(1, 328) = 1.50, *p* < .05] was related to higher MVPA participation rates for females (see Table 3). Descriptive chi square analyses indicated that when temperatures were above 90 degrees Fahrenheit, girls were more likely to participate in indoor PA games [adjusted standardized residual (ASR) = 4.0] than outdoor activities (asr = −2.7) where boys were equally likely to participate in outdoor (ASR = 3.8) as indoor PA (ASR = 5.5) during these exceptionally warm days [*χ*^2^ (4, 328) = 29.40, *p* = .000]. Physical features of the camp setting were not predictive of PA when examined across the full sample or for males’ PA specifically.

**Table 3:** Mean rate of moderately to vigorously active youth by physical environment features of summer camps

	**Total**^**a**^			**Males**^**b**^			**Females**^**c**^		
**Variable**	**Mean Obs/Unobs (SD)**	**F**	**ω**^**2**^	**Mean Obs/Unobs (SD)**	**F**	**ω**^**2**^	**Mean Obs/Unobs (SD)**	**F**	**ω**^**2**^
Supervision	2.12/1.95 (.22/.81)	0.05	.003	1.67/1.29 (.19/.67)	0.32	.002	.45/.66 (.10/.36)	0.30	.002
Equipment	2.46/1.61 (.49/.46)	4.38*	.010	1.71/1.25 (.40/.38)	1.87	.003	.75/.36 (.22/.21)	4.53*	.010
Temperature^d^	--	1.23	.020	--	1.00	.000	--	1.50*	.043
R^2^ (adj R^2^)			.15 (.05)			.14 (.03)			.15 (.05)

*Note*. “Usable” and “Accessible” were removed from analyses due to zero variance.

* *p* < .05 **p < .01.

a. Adjusted for variations in MVPA by camp (Camp1: *M* = 2.43, *SD* = .58; Camp2: *M* =2.04, *SD* = ..65; Camp3: *M* = 1.13, *SD* = .55; Camp4: *M =* 2.54, *SD* = .77): F(3, 329) = 1.78, p = .15, ω^2^ = .007.

b. Adjusted for variations in MVPA by camp (Camp1: *M* = 1.75, *SD* = .48; Camp2: *M* =1.54, *SD* = .54; Camp3: *M* = .66, *SD* = .45; Camp4: *M = 1.98*, *SD* = .64): F(3, 329) = 2.04, p = .11, ω^2^ = .009.

c. Adjusted for variations in MVPA by camp (Camp1: *M* = .68, *SD* = .26; Camp2: *M* = .50, *SD* = .29; Camp3: *M* = .47, *SD* = .25; Camp4: *M = .57*, *SD* = .35): F(3,329) = 0.17, p = .91, ω^2^ = .007.

d. Temperature is a continuous variable and tested as a covariate in the model with *M* = 88.54, *SD* = 6.57.

#### Social-motivational context

Assessment of social-motivational context (climate) features indicated that all four camps were highly supportive of youth autonomy, with 66% of the observations consisting of some type of autonomous activity (see Table 2). However, the majority of autonomous instances (89%) occurred during free play activities and did not involve the guided autonomy typically needed for fostering intrinsic motivation as specified by SDT. Across camps, 25% of the observations included highly engaging activities, and 24% of the observations involved activities with clearly defined rules and/or where youth participants understood what was expected of them (clarity of rules). Positive interactions between youth during PA were observed in 14% of the scans and 20% of the camp activities observed included PA games that were inclusive of all campers (e.g., involved teamwork, the majority of youth participated). Minimal organized activity was observed across camps (27%) and little-to-no bullying (.3%).

Chi Square analyses indicated significant variations between camps in the likelihood of observing clear rules [*χ*^2^ (3,329) =31.97, *p* = .000], autonomy support [*χ*^2^ (3,329) =23.16, *p* = .000], organized PA [*χ*^2^ (3,329) = 26.77, *p* = .000], and positive youth interactions [*χ*^2^ (3,329) = 15.60, *p* = .001]. The likelihood of observing motivational climate features primarily differed between low- and high-resourced camps (i.e., Autonomy, Organized PA, Positive Youth Interactions), with the likelihood of clarity of rules the only motivational climate feature that differed between low-resourced camps (see Table 2).

An ANOVA model which included all social-motivational predictors and controlled for variations by camp indicated that highly engaging games [F(1, 329) = 17.68, *p* < .001], positive peer interactions [F(1, 329) = 8.43, *p* < .01], and bullying [F(1, 329) = 9.39, *p* < .01] were significantly related to higher PA participation rates, and clarity of rules [F(1, 329) = 11.12, *p* < .001] was related to fewer youth participating in MVPA across the sample (see Table 4). Separate analyses for males and females indicated some sex differences in which climate features were related to PA. Although clarity of rules was related to less PA for both males [F(1, 329) = 7.54, *p* < .01] and females [F(1, 329) = 4.86, *p* < .05], highly engaging games [F(1, 329) = 23.10, *p* < .001] and bullying [F(1, 329) = 10.00, *p* < .01] was related to more PA for males but not females. In contrast, positive peer interactions was related to higher rates of PA for only females [F(1, 329) = 9.58, *p* < .01].

**Table 4:** Mean rate of moderately to vigorously active youth by summer camp social climate features

	**Total**^**a**^			**Males**^**b**^			**Females**^**c**^		
**Variable**	**Mean Obs/Unobs (SD)**	**F**	**ω**^**2**^	**Mean Obs/Unobs (SD)**	**F**	**ω**^**2**^	**Mean Obs/Unobs (SD)**	**F**	**ω**^**2**^
Clear Rules	6.22/8.0 (1.50/1.42)	11.12***	.025	4.97/6.20 (1.23/1.16)	7.54**	.016	1.25/1.83 (.73/.69)	4.86*	.011
Autonomy Support	7.19/7.06 (1.50/1.41)	0.06	.002	5.78/5.39 (1.24/1.16)	.88	.000	1.41/1.68 (.76/.69)	1.16	.000
Highly Engaging	8.00/6.26 (1.45/1.45)	17.68***	.042	6.40/4.77 (1.19/1.19)	23.10***	.055	1.59/1.49 (.71/.71)	0.26	.002
Inclusive	7.01/7.24 (1.47/1.44)	0.26	.002	5.46/5.71 (1.21/1.18)	0.47	.001	1.55/1.53 (.72/.70)	0.01	.003
Positive Interactions	7.83/6.42 (1.48/1.43)	8.43**	.018	5.92/5.25 (1.22/1.18)	2.86	.005	1.91/1.18 (.72/.70)	9.58**	.025
Bullying	11.42/2.83 (2.82/.27)	9.39**	.021	9.23/1.94 (2.32/.22)	10.00**	.022	2.19/.90 1.34/.13)	0.90	.000
Organized Activity	7.44/6.81 (1.48/1.42)	2.36	.003	5.78/5.39 (1.22/1.17)	1.39	.000	1.66/1.43 (.72/.69)	1.35	.001
R^2^ (adj R^2^)			.21 (.18)			.21 (.18)			.07 (.04)

* *p* < .05 **p < .01 ***p < .001.

a. Adjusted for variations in MVPA by camp (Camp1: *M* = 6.64, *SD* = 1.45; Camp2: *M* =7.34, *SD* = 1.46; Camp3: *M* = 6.84, *SD* = 1.46; Camp4: *M =* 7.59, *SD* = 1.48): F(3, 329) = 2.10, p = .100, ω^2^ = .008.

b. Adjusted for variations in MVPA by camp (Camp1: *M* = 5.17, *SD* = .1.19; Camp2: *M* =5.78, *SD* = 1.21; Camp3: *M* = 5.25, *SD* = 1.20; Camp4: *M = 6.14*, *SD* = 1.22): F(3, 329) = 2.96, p < .05, ω^2^ = .015.

c. Adjusted for variations in MVPA by camp (Camp1: *M* = 1.47, *SD* = .71; Camp2: *M* =1.65, *SD* = .72; Camp3: *M* = 1.59, *SD* = .71; Camp4: *M = 1.45*, *SD* = .72): F(3,329) = 0.35, p = .792, ω^2^ = .006.

#### Staff behaviors

Observations across camps indicated that staff were consistently present and interacted with the campers regularly throughout each day (31% of observations included general interactions between staff and campers unrelated to PA). In fact, there were very few instances where staff were observed engaging in behaviors that were not focused on the campers and their wellbeing (e.g., reading newspaper, talking on cell phone; 2.3%). However, we observed minimal interaction related to promoting youth physical activity (see Table 2). The most common staff behavior was “observing” physical activity (56%), followed by demonstrating or participating in the physical activity with the campers (10%). We observed very few verbal cues to initiate, increase, or praise physical activity (2%). Chi Square analyses indicated that the likelihood of “observing PA” was the only staff behavior that significantly varied between camps [*χ*^2^ (3,310) = 18.95, *p* = .000], where instances of staff observing were underrepresented in a low-resourced camp (Camp 1) and overrepresented in a high-resourced camp (Camp 4).

An ANOVA model that included all measured staff behaviors and controlled for variations by camp indicated that “observing youth” was the only staff behavior related to youth MVPA [F(1, 310) = 6.55, *p* < .05]. Separate analyses for males and females suggest that staff observing youth activity was significantly related to males’ higher rates of PA [F(1, 310) = 4.57, *p* < .05] but not females’ PA (see Table 5).

**Table 5:** Mean rate of moderately to vigorously active youth by summer camp staff behaviors

	**Total**^**a**^			**Males**^**b**^			**Females**^**c**^		
**Variable**	**Mean Obs/Unobs (SD)**	**F**	**ω**^**2**^	**Mean Obs/Unobs (SD)**	**F**	**ω**^**2**^	**Mean Obs/Unobs (SD)**	**F**	**ω**^**2**^
Promote PA	5.53/5.14 (1.72/1.70)	.07	.003	5.63/4.48 (1.41/1.39)	.96	.000	<.01/.66 (.84/.83)	1.18	.000
Increase PA	7.47/3.20 (2.98/.94)	1.84	.003	7.17/2.94 (2.45/.77)	2.67	.005	.30/.26 (1.46/.46)	.00	.003
Other Task	5.98/4.69 (1.83/1.44)	1.36	.004	5.72/4.39 (1.50/1.18)	2.15	.003	.26/.30 (.90/.71)	.01	.003
Participate/Demonstrate	5.60/5.07 (1.63/1.52)	.91	.003	5.29/4.82 (1.34/1.25)	1.06	.000	.31/.25 (.80/.74)	.051	.003
Observe	5.79/4.88 (1.59/1.53)	6.55*	.017	5.37/4.74 (1.31/1.26)	4.57*	.011	.42/.14 (.78/.75)	2.68	.005
R^2^ (adj R^2^)			.07 (.04)			.09 (.07)			.02 (.00)

* *p* < .05 **p < .01 ***p < .001.

a. Adjusted for variations in MVPA by camp (Camp1: *M* = 4.96, *SD* = 1.59; Camp2: *M* =6.04, *SD* = 1.56; Camp3: *M* = 4.68, *SD* = 1.58; Camp4: *M =* 5.67, *SD* = 1.58): F(3, 329) = 3.64, p = .013, ω^2^ = .024.

b. Adjusted for variations in MVPA by camp (Camp1: *M* = 4.74, *SD* = 1.30; Camp2: *M* =5.52, *SD* = 1.28; Camp3: *M* = 4.37, *SD* = 1.29; Camp4: *M = 5.59*, *SD* = 1.30): F(3, 329) = 4.80, p < .05, ω^2^ = .035.

c. Adjusted for variations in MVPA by camp (Camp1: *M* = .22, *SD* = .78; Camp2: *M* .51=, *SD* = .77; Camp3: *M* = .31, *SD* = .77; Camp4: *M = .*08, *SD* = .78): F(3,329) = 1.13, 2p = .336, ω^2^ = .001.

## Discussion

To our knowledge, this is the first study to conduct systematic observations of the physical and social-motivational resources of summer day camp settings and contributes to our understanding of the strengths and needs of summer camp programs to effectively promote youth PA in both boys and girls. Of the three major camp components assessed in the current study (physical, social climate, staff behaviors) the social climate features were most predictive of youth PA. In particular, clarity of rules was a primary predictor of youth MVPA across males and females. However, contrary to what might be expected based on previous research [57,58], higher clarity led to less participation and likely captured some of the challenges previously found in other PA-based settings (e.g., physical education) [70,71] such as extended time allocated to management activities (e.g., explaining rules, dividing youth into teams) and games which do not facilitate children’s continuous PA (e.g., waiting for a turn, sitting when “out”).

Additionally, some sex differences found for what social climate features predict PA indicate camp settings may need to target different components of the climate to meet the needs of both males and females. For males, findings suggest that the quality of the game is a key component for promoting PA with highly engaging games, most of which involved a competitive edge and increased potential for bullying (in the form of bickering, name-calling, and mild altercations), the most prominent predictor of participation. These findings are reflective of previous research which found that males were more likely than females to have a motivational profile that is high in both ego (e.g., winning, performance) and task (mastery) orientation which, in turn, was predictive of males' high motivation towards physical activity [72-75]. For females, findings support previous research [20-25,29,76] which suggests that social support and peer relations are key motivators for girls’ participation in PA. Together, these preliminary findings suggest camp activities will be most effective for promoting both girls’ and boys’ MVPA when they require minimal management and involve continuous activity for all participants, provide optimal challenge (e.g., developmentally appropriate), and help foster social connections. Specifically, if camp curriculums were designed so that youth could choose from multiple types of PA opportunities that ranged in degree of competitiveness, focus on mastery/skill development, and promoted collaboration, teamwork, and friendship, then camp programs may be more likely to satisfy all campers’ motivational needs (e.g., ego and task orientations, social connectedness), and in turn, increase participation in PA. The small, yet significant physical-environmental effects of temperature and equipment for girls also suggest that activities offered indoors (which was most common when temperatures were high), and the provision (and perhaps equal access) of equipment may also be important considerations for promoting girls’ PA.

Although social-motivational climate features explained a modest proportion of the variability in males MVPA, they explained a minimal proportion of the variability in females MVPA, with significant relations having small effect sizes. More research is needed to identify mechanisms that motivate girls to participate in PA. The infrequent occurrence of particular climate features (e.g., guided autonomy) may be one reason for the minimal effects found for girls. For instance, although staff behaviors were not predictive of youth PA in our study, this in no way leads us to conclude that staff behaviors are not important. Rather, the lack of significant findings is likely due to the minimal PA-based interaction observed between staff and youth, and highlights the need for increased staff training and the establishment of PA-based standards/goals to help guide staff implementation of camp curriculum. Given the relevance of the social experience for females’ PA participation, improving staff involvement may be particularly effective for promoting increases in girls’ PA.

Albeit a small sample size, chi square analyses indicated camps shared more commonalities than differences in physical and motivational climate characteristics, suggesting that many of the initial findings of summer camps’ strengths and needs identified in the present study may be applicable across summer camp programs. However, it is also important to note that significant variations that were found between camps primarily existed between high- and low-resourced camps, highlighting some of the challenges faced by camps with resource disparities, and perhaps, contributes to the explanation for why there were higher rates of MVPA found in high-resourced camps than low- resourced camps. For example, all camps had access to a gymnasium, however the limited size of Camp 2’s gymnasium made it more challenging to include all campers in any given activity. In response to this limitation, Camp 2 offered the most organized activities of all the camps where they could more effectively manage the allocation of space. Although this might be the most effective solution for promoting youth PA in these conditions, it may result in more idle time for youth (e.g., time spent in management, waiting for one’s turn) compared to youth in camps which did not have these space restrictions. Further research is needed to explore the various ways in which access to resources (e.g., removable and stationary equipment, number of paid staff, staff salary) influences camp design/curriculum and implementation for providing youth PA opportunities.

This study has several limitations. The current set of analyses test for the unique variance explained for each predictor in the model, however the contextual predictors measured in the present study are likely to function in interaction with one another to promote or inhibit youth PA [74,75,77-79]. Furthermore, the observation method used in the current study assumes that all youth within the specified area are exposed to the same climate components observed, however it is possible that observed components can be directed towards specific youth and not to others (e.g., staff interaction may be only directed towards the boys playing basketball and not to the girls sitting on the sidelines). However, both of these limitations reduce the likelihood of detecting significant effects, making this study’s findings particularly robust. Modifications to the method so that observations are conducted on specified individuals (similar to observational methods used in SOFIT) [68], and consideration of the interactive nature of camp features through the use of cluster analytical techniques, will likely increase the predictive value of the study’s specified climate components.

There are also a number of limitations associated with the application of a newly developed observational tool. For instance, there are likely to be additional important physical and social motivational factors within the camp setting for promoting youth PA that are not currently included in the MCOT-PA. For example, we anticipate that youth MVPA may vary by the extent to which the PA options offered in the setting are competitive (vs. mastery or task-oriented). The traditional versions of many competitive PA games are structured in a way that campers have to wait on the sidelines for their turn or can be eliminated from the game in an unsuccessful round, resulting in higher levels of sedentary behavior and possible discouragement and disengagement from the activity altogether. Similarly, the age of the youth observed, and the developmental appropriateness of the activities offered have been shown to be important predictors of youth MVPA in previous research [80]. Future MCOT-PA assessments will include a “competitiveness” construct and a “developmentally appropriate” construct in order to examine the nature of these context features for promoting youth MVPA. To assess differences by age, future research will also need to either employ additional methods which link individual data to observations (similar to SOFIT method) [68], or divide youth by age group prior to observation. Future research should also assess variations in youth responses to context features by other key intrapersonal characteristics predictive of youth PA such as weight status and PA self-efficacy.

Although the present study provides initial indications of adequate inter-observer reliability and predictive validity of the MCOT-PA, given this is the first study to use the MCOT-PA, additional research is needed to provide further support for the psychometric properties of the observational tool. The minimal instances of staff behaviors observed in the present study raised particular concerns that either the staff constructs included in the observation tool, or some feature of the data collection protocol implemented in the present study, are not adequate for capturing staff behaviors within camp settings. Given previous observation studies have demonstrated predictive validity of the staff constructs included in the present study [57], in future work we will first test whether reducing the time lapse between observations from 15 minutes (the suggested protocol in the SOPLAY manual) to a continuous observational cycle (in which one observation immediately follows the previous observation), will better capture the nature of staff behaviors.

Lastly, where the methods used in this study provide the first detailed overview of the camp climate and its relation to youth behaviors, in no way can we infer causality. Implementation of a social-environmental intervention within camps which addresses the key components identified in this study will shed further light on the importance of the physical and social climate for promoting youth engagement in PA.

## Conclusion

Given the summer months represent the time of the year when the risk for youth weight gain is high [8], research on the extent to which camp settings provide important physical and social-motivational features for promoting PA among all participating youth is key for informing future intervention and youth summertime programming policy. Direct observation methods have been increasingly viewed as a highly effective technique for youth-based research [81], avoiding the limited accuracy of youth self-report [82-84] and the expense and participant burden of objective activity monitors [85,86]. Further, the momentary sampling techniques of the SOPLAY protocol used in the present study enables researchers to gather data on the PA of groups as it co-occurs with the (continuously changing) motivational climate of the context in which they are participating [81]. Although, there are observation tools which include an assessment of the physical environment, staff/teacher behaviors, and/or other basic contextual features (e.g., type of activity), to date, little-to-no observation tools have been designed to assess the motivational climate features of youth PA settings [61]. Findings from the present study indicate an assessment of the motivational climate of youth settings, such as summer day camps, contributes to our understanding of the contextual supports and barriers of youth PA within these settings. Identifying and then targeting change in key social-climate factors, will result in inducing relatively stable changes in target behaviors (e.g., PA), rendering youth programs consistently more effective.

With increased funding for new summer activities as part of the American Recovery and Reinvestment Act of 2009, we are at a critical point for informing the design and implementation of summer camps that promote youth health [87]. The findings of the present study, albeit a small sample, suggests that summer day camps can be a key antidote to the increases in youth sedentary behavior during the summer months. By nature, most camps provide opportunities for physical activity, but more can be done to encourage physical activity through improvements in staff training, program scheduling, and program activities at camp. Further research is needed to advance our understanding of what factors are essential for ensuring that we provide the healthiest and most physically active environments for children and adolescents at camp. Moreover, youth at greatest risk for obesity are also the least likely to have access (e.g., transportation, costs) to the health-promotion resources afforded by participation in summer day camps. Additional initiatives are necessary to increase access and affordability of quality summer camp programs to ensure all youth are provided the opportunities necessary for promoting healthy developmental trajectories.

## Abbreviations

PA: Physical activity; PYD: Positive youth development; SDT: Self-determination theory; SES: Socioeconomic status.

## Competing interests

The authors declare that they have no competing interests.

## Authors’ contributions

All authors assisted with drafting or revising the manuscript. NZ originated the idea of the study, and is responsible for the design of the social-motivational supplemental measurement tool and study design. All authors trained, piloted, and helped revise the measures and described study protocol. CS and BS contributed to data collection and data management. NZ supervised the study. All authors provided feedback on the manuscript and approved the final version.
